# Correction: Orbital entanglement and CASSCF analysis of the Ru–NO bond in a Ruthenium nitrosyl complex

**DOI:** 10.1039/c5cp90073e

**Published:** 2015-05-13

**Authors:** Leon Freitag, Stefan Knecht, Sebastian F. Keller, Mickaël G. Delcey, Francesco Aquilante, Thomas Bondon Pedersen, Roland Lindh, Markus Reiher, Leticia Gonzalez

**Affiliations:** a Institut für theoretische Chemie , Universität Wien , Währinger Str. 17 , 1090 Vienna , Austria . Email: leticia.gonzalez@univie.ac.at; b ETH Zürich , Laboratory of Physical Chemistry , Vladimir-Prelog-Weg 2 , 8093 Zürich , Switzerland . Email: markus.reiher@phys.chem.ethz.ch; c Department of Chemistry – Ångström , The Theoretical Chemistry Programme , Uppsala University , Box 518 , 751 20 Uppsala , Sweden; d Dipartimento di Chimica ‘‘G. Ciamician’’ , Università di Bologna , V. F. Selmi 2 , 40126 Bologna , Italy; e Centre for Theoretical and Computational Chemistry , Department of Chemistry , University of Oslo , P.O. Box 1033 Blindern , 0315 Oslo , Norway; f Uppsala Center for Computational Chemistry - UC3 , Uppsala University , Box 518 , 751 20 Uppsala , Sweden

## Abstract

Correction for ‘Orbital entanglement and CASSCF analysis of the Ru–NO bond in a Ruthenium nitrosyl complex’ by Leon Freitag *et al., Phys. Chem. Chem. Phys.*, 2015, DOI: ; 10.1039/c4cp05278a.

The authors would like to explicitly acknowledge the exchange funding of a European Short Term Scientific Mission (STSM) between Austria and Switzerland, and therefore the amended Acknowledgments should read as follows:

Financial support from the University of Vienna, the COST Action CM1305 ‘‘Explicit Control Over Spin-states in Technology and Biochemistry’’ (STSM 21153), the Research Council of Norway through a Centre of Excellence Grant (Grant No. 179568/V30), the Italian Ministry of Education and Research (MIUR)-Grant No. RBFR1248UI, the Uppsala University, eSSENCE, and the Swedish Research Council (VR) is gratefully acknowledged. Furthermore, we thank Yingjin Ma for the help with the MAQUIS-MOLCAS interface, as well as Vladimir Arion and Gabriel Büchel for fruitful discussions.

The Royal Society of Chemistry apologises for these errors and any consequent inconvenience to authors and readers.

